# Customizing the mechanical properties of additively manufactured metallic meta grain structure with sheet-based gyroid architecture

**DOI:** 10.1038/s41598-022-24207-4

**Published:** 2022-11-18

**Authors:** Kibeom Kim, Gun-hee Kim, Hyung Giun Kim, Hoe Joon Kim, Namjung Kim

**Affiliations:** 1 Molding & Metal Forming R&D Department, Korea Institute of Industrial Technology (Research Institute of Advanced Manufacturing & Materials Technology), Bucheon, 14441 South Korea; 2 Functional Materials and Components R&D Group, Korea Institute of Industrial Technology (Gangwon Division), Gangneung, 25440 South Korea; 3 Department of Robotics Engineering, Daegu Gyeongbuk Institute of Science and Technology (DGIST), Daegu, 42988 South Korea; 4 Department of Mechanical Engineering, Gachon University, Seongnam, 13120 South Korea

**Keywords:** Mechanical engineering, Software

## Abstract

The use of cellular structures has led to unprecedented outcomes in various fields involving optical and mechanical cloaking, negative thermal expansion, and a negative Poisson’s ratio. The unique characteristics of periodic cellular structures primarily originate from the interconnectivity, periodicity, and unique design of the unit cells. However, the periodicity often induces unfavorable mechanical behaviors such as a “post-yielding collapse”, and the mechanical performance is often limited by the design of the unit cells. Therefore, we propose a novel structure called a meta grain structure (MGS), which is inspired by a polycrystalline structure, to enhance flexibility in design and mechanical reliability. A total of 138 different MGSs were built and tested numerically, and the correlations between the design parameters (e.g., the relative density) and mechanical properties of the MGSs were rigorously analyzed. A systematic design methodology was developed to obtain the optimal design of the MGS with the target Young’s modulus. This methodology makes it possible to build a unique structure that offers various design options and overcomes the current limitations of cellular structures. Furthermore, a systematic inverse design methodology makes it possible to produce an MGS that satisfies the required mechanical performance.

## Introduction

A cellular structure is composed of an interconnected network of struts or surfaces that form the edges and faces of cells^1^. Recent advances in additive manufacturing technology have enabled numerous novel designs of cellular structures that were otherwise not possible to achieve owing to the limitations of traditional manufacturing processes. Depending on the materials and designs used, such cellular structures can yield unprecedented results such as a negative Poisson ratio^2^, negative thermal expansion^3^, optical metamaterials^4^, and ultralight structural parts with extremely high stiffness–weight ratios^5^. The large surface-to-volume ratios of cellular structures enables their use as acoustic insulators^6^ and heat exchangers^7^. Cellular structures have also been used as scaffolds in the field of biomedicine to maximize cell viability^8^.

Various design approaches with the assistance of computational resources have been proposed for cellular structures^9,10^ Cellular structures can be classified in terms of their geometrical configuration, deformation mechanism, and degree of order. Based on the degree of order, cellular structures can be classified as periodic or disordered structures^11^. A periodic cellular structure is composed of repeating unit cells. The various extraordinary characteristics of periodic cellular structures, such as their mechanical, electrical, and optical properties, primarily originate from the interconnectivity, periodicity, and unique design of the unit cell. For example, nature-inspired geometries such as honeycombs demonstrate outstanding mechanical strength with a relatively low density^12^. Man-made structures that mimic crystal structures in nature, such as a-cristobalite and LaNiO3/SrTiO3, exhibit ultrahigh strength and ductility^13^. Structures that are inspired by face-centered and vertex cubes show better mechanical properties than foam structures. In addition, the energy absorption efficiency of an edge-centered cube lattice is superior to that of other lattice structures^14^. However, recent studies have indicated that most cellular structures with repeating unit cells exhibit a “post-yielding collapse” because of shear bands^15^, which results in low energy absorption^16–18^. Furthermore, a cellular structure exhibits low connectivity if it is composed of randomly distributed polygons. Consequently, the structure has unstable support, resulting in an unfavorable mechanical performance.

A disordered cellular structure refers to a structure with randomly distributed unit cells, wherein the cells that constitute the foam structure have different sizes and shapes. The mechanical properties of a disordered cellular structure are governed by the geometry of the structure and mechanical properties of the base material. A disordered cellular structure often mimics the complex and anisotropic microstructures of natural materials such as bone tissues^19^. However, the mechanical properties of disordered cellular structures, such as the Young’s modulus and yield strength, decrease significantly as their relative density decreases because of their stochasticity, structural inhomogeneity, and bending-dominated deformation mode^1,20^. Because of the inherent limitations originating from the stochasticity of disordered cellular structures, a systematic design methodology has not yet been fully developed. Some of the design methodologies for disordered cellular structures based on random sampling lack repeatability in terms of the morphology and show significant uncertainties in terms of the material properties^21^.

Recently, hybrid cellular structures have been developed to overcome the limitations of periodic and disordered cellular structures. These structures enable the control of the geometric and mechanical parameters in a gradient manner. For example, Yang et al. proposed a heterogeneous lattice with a tunable stiffness by partitioning subspace and filling in each subspace with hierarchical substructures^22^. Ren et al. proposed a design methodology for a multi-scale lattice with multiple triply periodic minimal surfaces (TPMSs) by locating different lattice cells based on the stress regions. The stiffness and strength of the resulting hybrid structure were 31% and 21% higher than those of the traditional TPMS lattice, respectively^23^. Song et al. proposed a hybrid structure composed of simple cubic and body-centered cubic unit cells produced by utilizing a crystal twinning mechanism. This structure increased the stiffness, strength, and energy absorption by 50.82%, 10.94%, and 20.06%, respectively^24^. In addition, a functionally graded lattice structure was proposed to vary the relative density perpendicular to the loading direction. However, the relative densities of this structure changed layer by layer. Thus, the overall mechanical properties relied significantly on the layer with the lowest relative density^25,26^.

In this study, a hybrid structure called a meta grain structure (MGS) that could offer advantages of both periodic and disordered cellular structures was proposed. An MGS is defined as a hierarchical structure comprising multiple grains with a TPMS structure. The use of an MGS helps realize a wide feasible range of mechanical properties, including the extreme mechanical properties exhibited by periodic cellular structures. The design of an MGS is described in “Design of meta grain structure” section. “Finite element analysis with numerical homogenization” section provides the details of the numerical homogenization of the TPMS, which enhances the computational efficiency. A machine learning (ML)-based regression model and a systematic design methodology for the inverse design of an MGS are described in “ML-based regression models” and “Inverse design of MGS using particle swarm optimization (PSO)” sections, respectively. “Results and discussions” section discusses the generated Voronoi cells and feasible mechanical properties of MGSs and describes how the mechanical properties of an MGS can be described. It also explains the design of an MGS that satisfies a target mechanical property using the proposed design methodology, which is verified using a finite element simulation. “Conclusions” section concludes this paper and describes the scope for future work.

## Materials and methods

### Design of meta grain structure

The grains in the MGS were generated via a Voronoi tessellation, which is one of the most widely used methods for generating grains because of its simplicity and efficiency^27^. The seeding points for the Voronoi tessellation were sampled from Poisson’s probability distribution (PPD). The PPD exhibits the following unique characteristics that are suitable for generating an MGS: (i) the seeding points are generated independently from the previous iterations; (ii) the probability of a seeding point being generated in a space is proportional to the size of the space; and (iii) the probability of any two seeding points being generated at the same location converges to zero^28,29^. In addition to the PPD, the SSI algorithm was implemented to regulate the average size of the grains^30^. The SSI algorithm eliminates a generated point if the distance between the previous and generated seeding points is within the inhibition distance. This helps reduce uncertainties that arise from insufficient sampling and ensure that the average grain structure of the MGS is in a specific range. In this study, the source code libraries that are available in the public domain were used and modified for our research purpose.

The TPMS structure was placed inside each grain to enhance the mechanical properties of the MGS and enable design flexibility. A sheet-based gyroid was selected because of its robustness to any geometrical uncertainties that might be generated during manufacturing^31^ and its superior mechanical properties compared with those of a skeletal-based gyroid^20^. The commercial solid modeling software SpaceClaim in ANSYS 2020 R1 (ANSYS, USA) was used to develop the sheet-based gyroid model.

### Finite element analysis with numerical homogenization

A three-dimensional finite element (FE) model was developed to evaluate the mechanical behavior of the MGS under compressive loading. The analysis was performed using the commercial software ABAQUS standard 6.14 (Dassault Systèmes, France). The 10-node quadratic-tetrahedron-type element was used to simulate the deformation behavior of the MGS under compression in the linear elastic regime.

**Figure 1:**
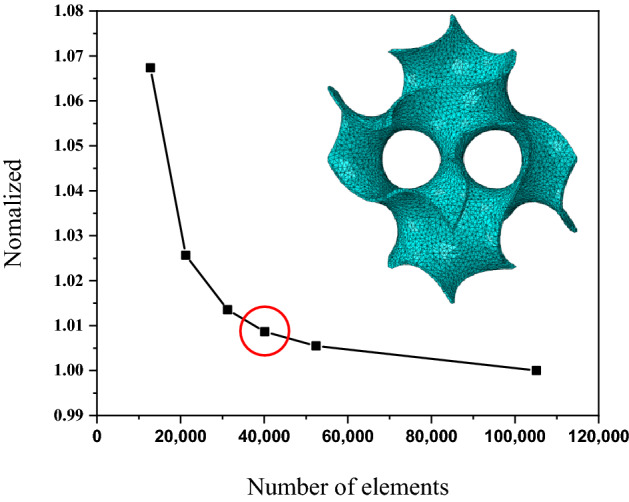
Mesh convergence study performed for gyroid structure. The result indicates the optimum mesh size for the single gyroid structure (40,216 elements) without sacrificing the computational accuracy.

A mesh convergence study was performed to determine the most suitable element size for efficient simulations without any reduction in accuracy. The specimen was a cube with a length of 6.25 mm, which was composed of repeating sheet-based gyroids. The nodes at the top surface had a displacement of 0.25 mm in the downward direction, while the bottom surface was fixed. It was assumed that the MGS was made of commercially pure titanium (CP-Ti) and fabricated using a selective laser melting (SLM) process, so that the Young’s modulus and Poisson’s ratio of the CP-Ti were set at 115 MPa and 0.34, respectively^32^. Six different numbers of elements (12,777; 21,240; 31,242; 40,126; 52,406; and 105,129) were tested to determine how the mechanical properties varied with the mesh size. The results are presented in Fig. 1, where the X-axis represents the number of elements, and Y-axis represents the normalized stress. As the number of elements increased, the normalized stress converged to a certain value. In this study, we selected 40,126 elements because the deviation between the results obtained using 40,126 and 105,129 elements was within 1%. This result confirmed that further analyses would be insensitive to the mesh size.

To estimate the mechanical properties of the sheet-based gyroid as a function of the relative density, its deformation behavior under compression loading was simulated. The FE model comprised 4 × 4 × 4 sheet-based gyroids, where the thickness of the sheet-based gyroids changed from 0.1 to 0.5 mm. The simulation setup was similar to that used in the mesh convergence study except for the top surface displacement, which was 1.25 mm.

**Figure 2:**
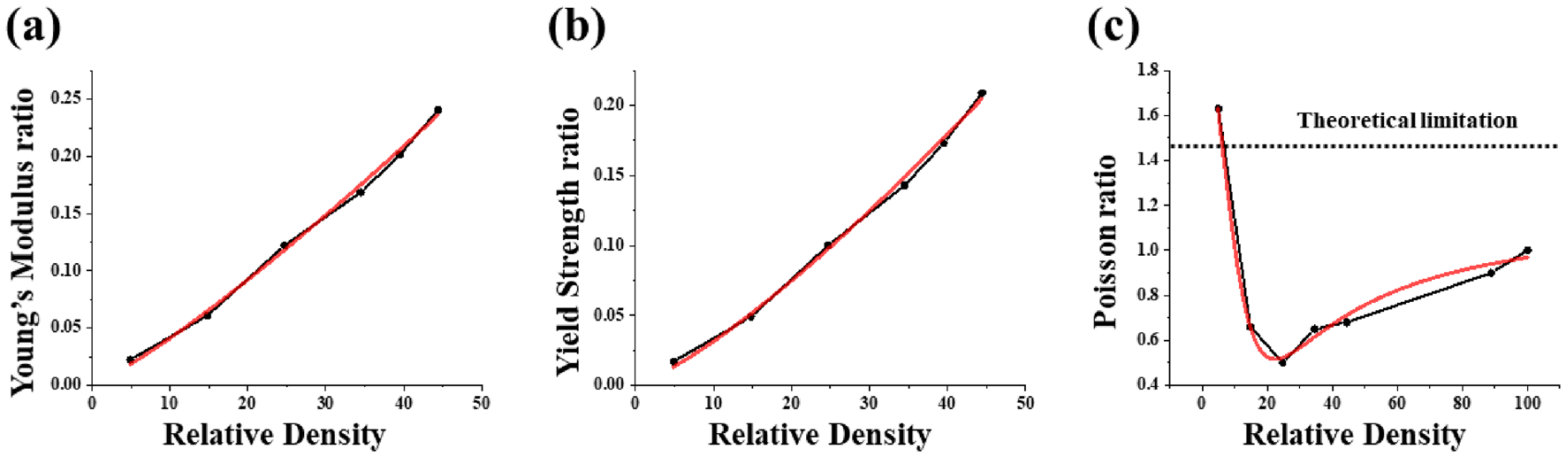
Mechanical properties of the gyroid as a function of relative density: (**a**) young’s modulus, (**b**) yield strength, (**c**) normalized Poisson’s ratio. The dotted line in (**c**) displays the theoretical limitation.

MATLAB (Mathworks, USA) code was developed in-house to extract the Young’s modulus and yield strength from the stress–strain curve. The Gibson–Ashby model in the form of an exponential equation with two coefficients^33^ was used to extract the relationship between the relative density of the sheet-based gyroid and the mechanical properties. These coefficients were estimated using the simulation data substituted into Eqs. (1)–(3). The results are shown in Fig. 2.

1 $${E}_{L}={E}_{B}{C}_{1}{({\rho }^{*})}^{n}=\left(107.0\right)\times \left(1.61\right)\times {({\rho }^{*})}^{1.277},$$

2 $${\sigma }_{L}={\sigma }_{B}{C}_{2}{({\rho }^{*})}^{m}=\left(626.1\right)\times \left(2.586\right)\times {({\rho }^{*})}^{1.189},$$

3 $${\upsilon }_{L}=\left[\frac{{p}_{1}{\left({\rho }^{*}\right)}^{2}+{p}_{2}\left({\rho }^{*}\right)+{p}_{3}}{{\left({\rho }^{*}\right)}^{2}+{q}_{1}\left({\rho }^{*}\right)+{q}_{2}}\right]=\left[\frac{0.4069 \times {\left({\rho }^{*}\right)}^{2}-12.206 \times \left({\rho }^{*}\right)+152.777}{{\left({\rho }^{*}\right)}^{2}-10.568\left({\rho }^{*}\right)+213.144}\right].$$

Here, ${E}_{L}$ and ${E}_{B}$ are the Young’s modulus values (unit: GPa) for the sheet-based gyroid and base material, respectively, and ${\sigma }_{L}$ and ${\sigma }_{B}$ are the corresponding yield strengths (units: MPa). The ${E}_{B}$ and ${\sigma }_{B}$ values were obtained from Ref.^32^. ${C}_{1}$ and ${C}_{2}$ are proportionality constants specific to the material, and $n$ and $\text{m}$ are exponential coefficients based on the mechanical behavior of the cellular structure. ${\rho }^{*}$ is the relative density of the sheet-based gyroids. To obtain the Poisson’s ratio as a function of the relative density, FE analyses were performed in three different loading directions, i.e., along the X-, Y-, and Z-axes. The loading exerted along the Z-axis deformed the specimen in the X- and Y-directions; therefore, the average strain values in these two directions were calculated to obtain the Poisson’s ratio. A rational function comprising five coefficients was selected for the best fit.

Numerical homogenization was employed to increase the calculation efficiency. This method can efficiently extract the effective properties of a lattice structure comprised of sheet-based gyroids. After homogenization, the mechanical properties of each grain, denoted as “${G}_{i}$”, can be defined by the relative density. In this study, the relative density for the grains ranged from 7 to 70% of that for the bulk material to reduce the computational complexity. A total of 138 cases of numerical homogenization were used to build the database (Supplement data).

### ML-based regression models

To maximize the efficiency of the calculation, a regression model was implemented to predict the Young’s modulus of the MGS. Four different ML-based models (decision tree, boosting tree ensemble, support vector machine (SVM), and Gaussian process regression (GPR)) in the MATLAB Regression Learner App (Mathworks, USA) were employed to predict the relationship between the design parameters and Young’s modulus of the MGS^34^. The minimum leaf size of the decision tree was set to four, and the degree of the polynomial of the SVM was set to two. In the boosting tree ensemble model, the minimum leaf size and number of learners were set to 8 and 30, respectively. The learning rate of the boosting tree ensemble model was 0.1. The Matern 5/2 covariance function was used for the GPR.

### Inverse design of MGS using particle swarm optimization (PSO)

Finding the design parameters for an MGS that satisfies the target Young’s modulus can be considered an optimization problem that can be formulated as follows:

4 $$\underset{x\in X}{\text{max}}[f\left(x\right)],$$

where $x=({\rho }_{1},{\rho }_{2},\dots ,{\rho }_{n})$ is a vector of the design parameters, i.e., the density of each grain of the MGS in an n-dimensional design space, X. In addition, n is the number of grains, and $f$ is the objective function, i.e., the Young’s modulus of the MGS.

PSO was used to determine the design parameters for an MGS that satisfied the target property. The PSO defined a vector for the design parameters, ${x}_{i}$, as an individual ith agent and distributed all the agents randomly in the decision space. Each agent navigated the decision space based on its experiences with its neighbors as follows:

5 $${\underline{v}}_{i}\text{(t) = k}{\underline{v}}_{i}\text{(t-1) + }{C}_{3}{r}_{1}\text{(}{\underline{x}}_{pbest,i}-{\underline{x}}_{i}\text{(t)) + }{C}_{4}{r}_{2}\text{(}{\underline{x}}_{leader}-{\underline{x}}_{i}\text{(t))},$$

6 $${\underline{x}}_{i}\text{(t) = }{\underline{x}}_{i}{({\text{t}}-1) + }{\underline{v}}_{i}\text{(t)},$$

where k is the weight coefficient that models the inertia of the agent and affects the dependence of the current velocity on the previous velocity. Here, t represents the iteration step; ${\underline{x}}_{pbest,i}$ and ${\underline{x}}_{leader}$ correspond to the best cost function values for the ith agent and leader, respectively. The leader is defined as the agent with the best cost function value at a certain iteration. ${C}_{3}$ and ${C}_{4}$ are the coefficients corresponding to the effects of the personal best experience and the neighbor’s best experience pertaining to the velocity, respectively; ${r}_{1}$ and ${r}_{2}$ are random values sampled from a uniform distribution in [0, 1]. Utilizing a random value as a coefficient increases the robustness and randomness of the algorithm. After identifying the location of each agent after a certain iteration in the decision space, the estimated regression model calculates the cost function corresponding to each agent. As the iteration proceeds, the agent evolves and converges toward the leader. The leader is replaced when the agent has a better cost function value than the current leader. This procedure is repeated until all the agents reach the global optimizer.

## Results and discussions

### Voronoi tessellation with SSI and PPD for meta grains

**Figure 3:**
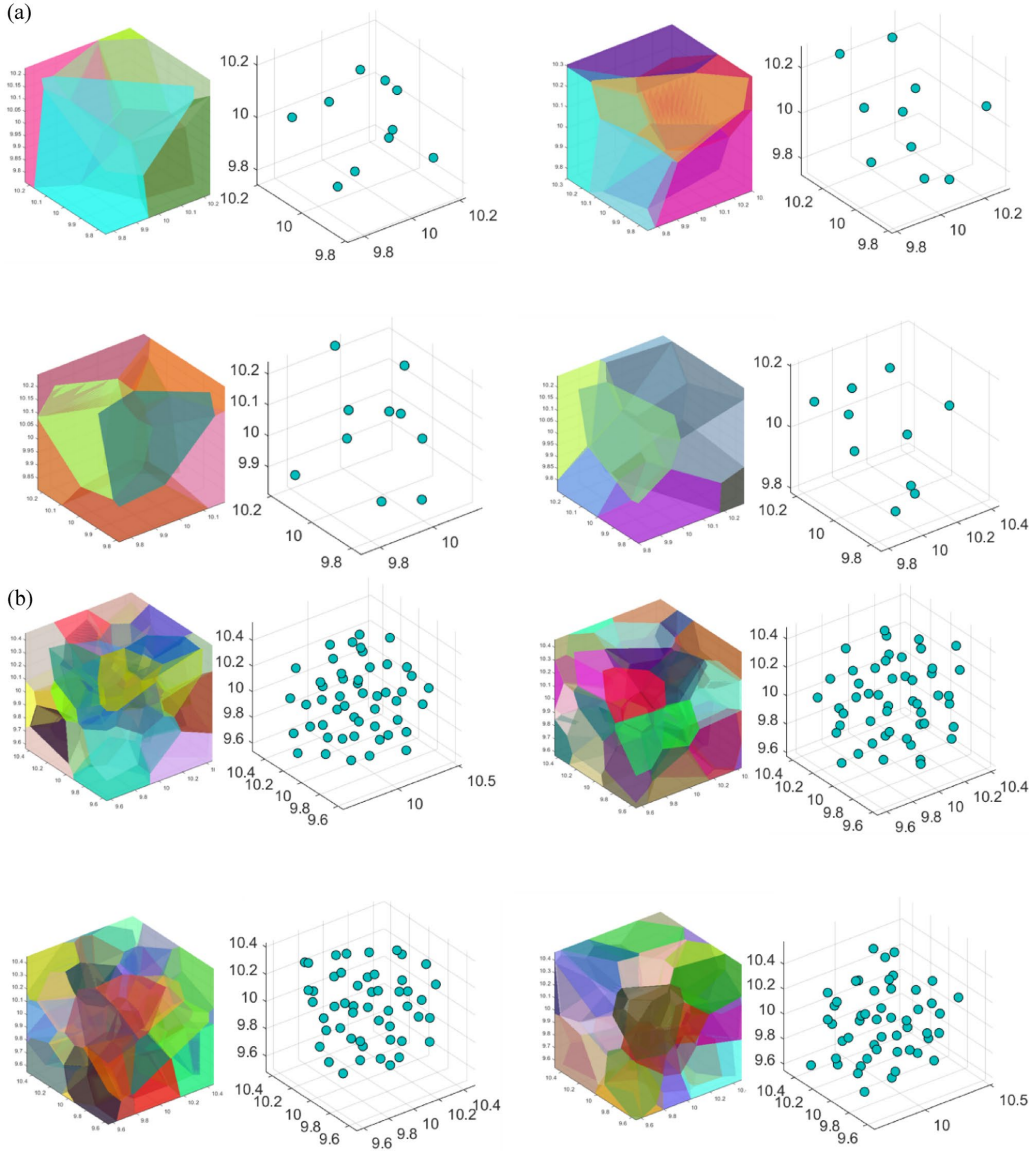
Representative Voronoi cells with (**a**) 10 and (**b**) 50 seeding points produced by the proposed algorithm.

**Figure 4:**
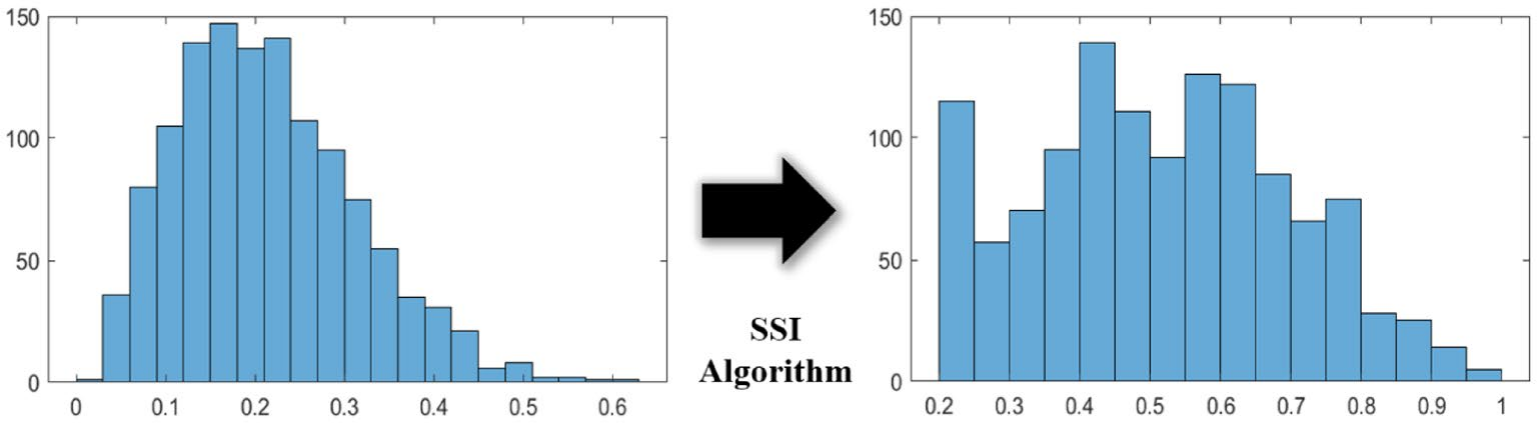
Effect by sequential spatial inhibition (SSI) algorithm.

The Voronoi-based porous structure was used to build the meta grains. The design space was divided into individual Voronoi cells based on the given seed points. By controlling the density of seeding points in space, a uniform or graded porosity distribution could be generated. In this study, seed points sampled from the PPD were used to generate isotropic meta grains. Figure 3a,b show the representative Voronoi cells produced by the proposed algorithm when the number of seeding points is 10 and 50, respectively. As the number of seeding points increases, the uniformity of the grain distribution increases. This implies that a small number of seeding points can easily be affected by insufficient sampling. Therefore, the SSI algorithm was implemented to reduce the effect of insufficient sampling. Figure 4 shows the effect of the SSI algorithm on the distribution of the relative distances between seeding points. By eliminating the generated point within the inhibition distance, this algorithm increases the uniformity of the distribution of Voronoi cells. Table 1 lists the number of grains, average grain volume, and size of four Voronoi cells with four individual samplings. The results indicated the repeatability of the Voronoi cells generated using the PPD and SSI algorithms.

**Table 1:** Number of grains, average grain volume and size of Voronoi cells from individual samplings.

Case	Number of grains (ea)	Average grain volume (mm^3^)	Average grain size (mm)
1	48	312.50	18.06
2	47	312.50	18.06
3	47	305.91	17.93
4	47	311.97	18.05

### Feasible mechanical properties of MGS

**Figure 5:**
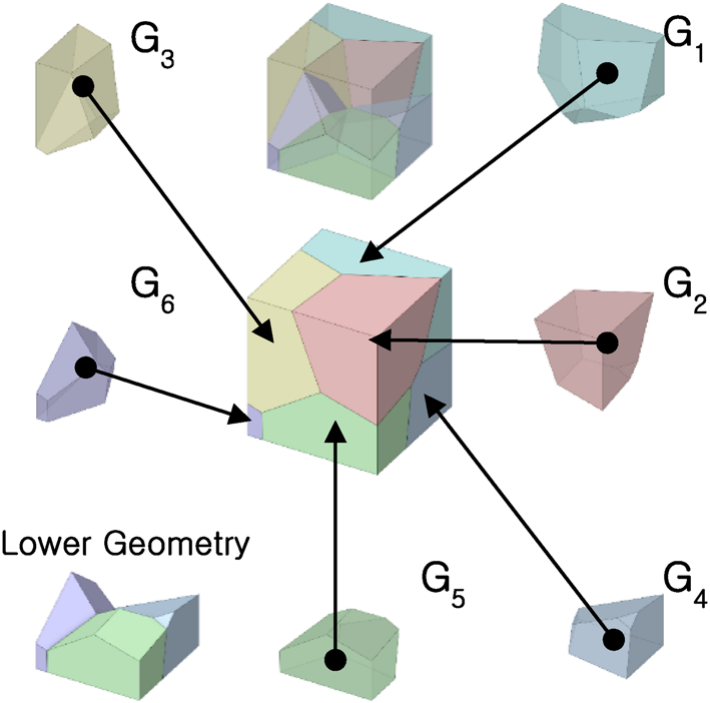
MGS comprises of 6 Voronoi cells as a test sample.

**Figure 6:**
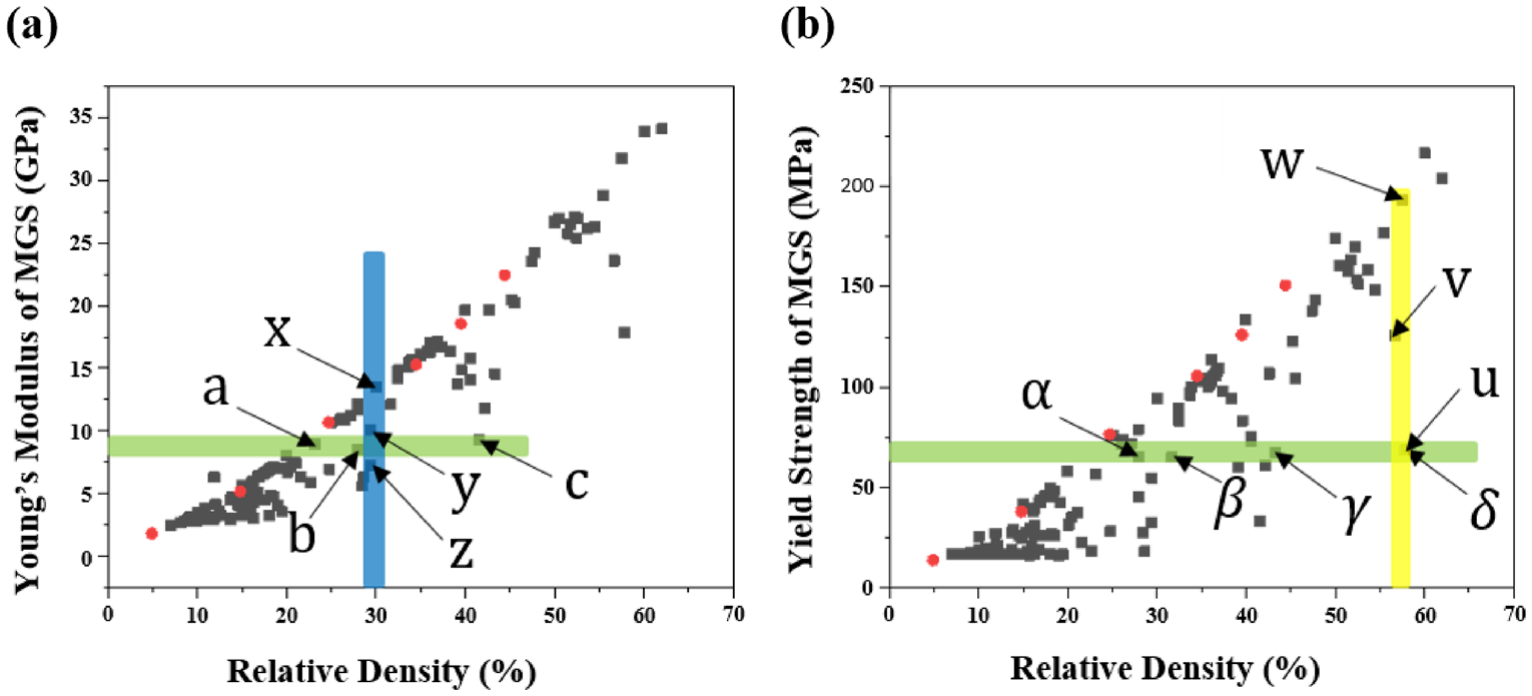
Feasible mechanical properties of MGS with respect to relative density: (**a**) young’s modulus, (**b**) yield strength with respect to relative density. The black dots are originated from 138 MGS structures and the red dots represents the mechanical properties of the repeating sheet-based gyroid.

In this study, an MGS with six grains was created as an example to demonstrate the mechanical behavior of MGSs, as shown in Fig. 5. Each grain was labeled ${G}_{i}$, where i = 1, 2,…,6. Sheet-based gyroids with specific relative densities were placed in each grain. A total of 138 FE analysis models were constructed to build a database containing the mechanical properties of MGSs with different relative densities. The black points in Fig. 6a,b represent the feasible Young’s modulus and yield strength of the MGS, respectively. The red points indicate the relationship between the relative density and mechanical properties of the repeating sheet-based gyroids. Compared with the wide feasible area encompassed by the MGS, the relationship between the relative density and mechanical properties of the repeating sheet-based gyroids could be regressed to a single line. This implied that if the relative density of the repeating-sheet-based gyroids is determined, then the mechanical properties will be fixed. This strong correlation between the mechanical property and relative density is a common design limitation when a repeating unit cell is used. Therefore, the proposed MGS offers a wide feasible area of mechanical properties, thereby affording design flexibility.

### Independent control of Young’s modulus of MGS with respect to density

**Figure 7:**
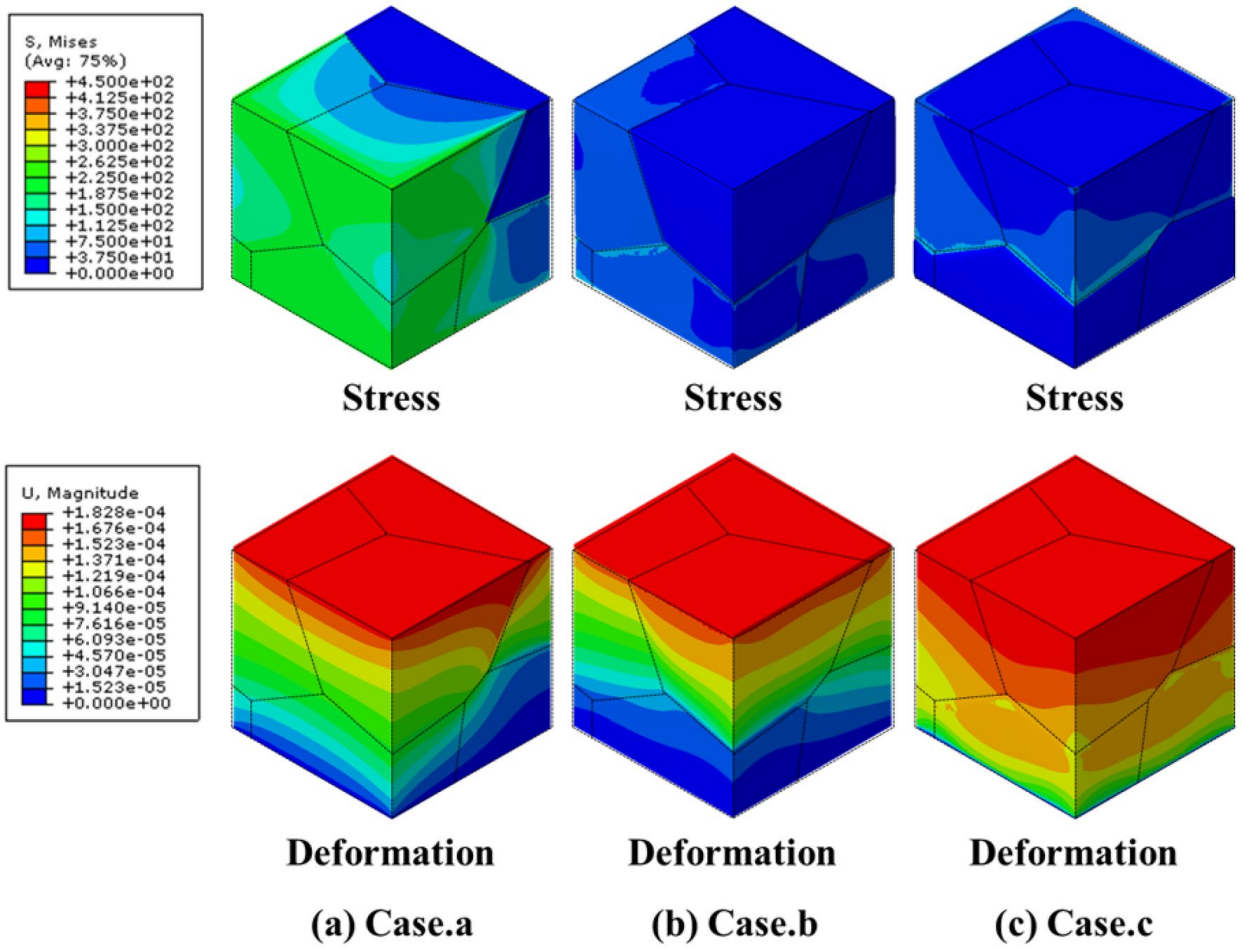
Stress and deformation FEM results of group 1.

The two groups highlighted in Fig. 6a were selected to comprehensively explain the independent control of the Young’s modulus of the MGSs. The relative density and Young’s modulus of each MGS are listed in Table 2, and the specific relative densities of all grains are listed in Table 3. ${G}_{Total}$ in Table 3 represents the relative density of the MGS, which was calculated based on the relative densities of ${G}_{1}-{G}_{6}$ and the corresponding grain volumes. In group 1, all the points had similar Young’s modulus values in the range of 8.55–9.27 GPa, but the relative densities ranged broadly from 23.20 to 41.55%. This implied that the relative density of the MGS could be controlled independently while maintaining a fixed Young’s modulus. For case “a”, all the relative densities of the grain structure were 30% except ${G}_{1}$, which had a value of 7%. Hence, the mechanical properties of case “a” were similar to those of the 30% sheet-based gyroids, but the mechanical properties of the MGS deteriorated because of ${G}_{1}$, as shown in Fig. 7a. For case “b”, ${G}_{3}$ was the strongest among the three upper grains (${G}_{1}$–${G}_{3}$), whereas ${G}_{5}$ and ${G}_{6}$ were the strongest among the lower grains. Therefore, in case “b”, ${G}_{3}$, ${G}_{5}$, and ${G}_{6}$ formed the backbone of the entire structure. Thus, the Young’s modulus was a function of the relative densities of these grains, as shown Fig. 7b. For case “c”, the three upper grains exhibited a relative density of 10%, whereas the three lower grains exhibited a relative density of 60%. Therefore, case “c” exhibited the highest total relative density, and its mechanical properties were similar to those of the other cases because of the upper structure, as shown in Fig. 7c.

**Table 2:** Relative densities and the corresponding Young’s moduli of MGSs in Group 1 and Group 2.

Properties	a	b	c	x	y	z
Relative density (%)	15.94	27.95	41.55	30	29.41	29.44
Young’s modulus (GPa)	8.99	8.55	9.27	13.55	10.12	7.26

**Table 3:** Relative densities of MGS in Group1 and Group 2.

Case	${G}_{1}$ (%)	${G}_{2}$ (%)	${G}_{3}$ (%)	${G}_{4}$ (%)	${G}_{5}$ (%)	${G}_{6}$ (%)	${G}_{Total}$ (%)
a	7	30	30	30	30	30	15.94
b	10	10	30	30	60	60	27.95
c	60	60	60	10	10	10	41.55
x	30	30	30	30	30	30	30
y	10	20	30	40	50	60	29.41
z	10	60	10	60	10	60	29.44

**Figure 8:**
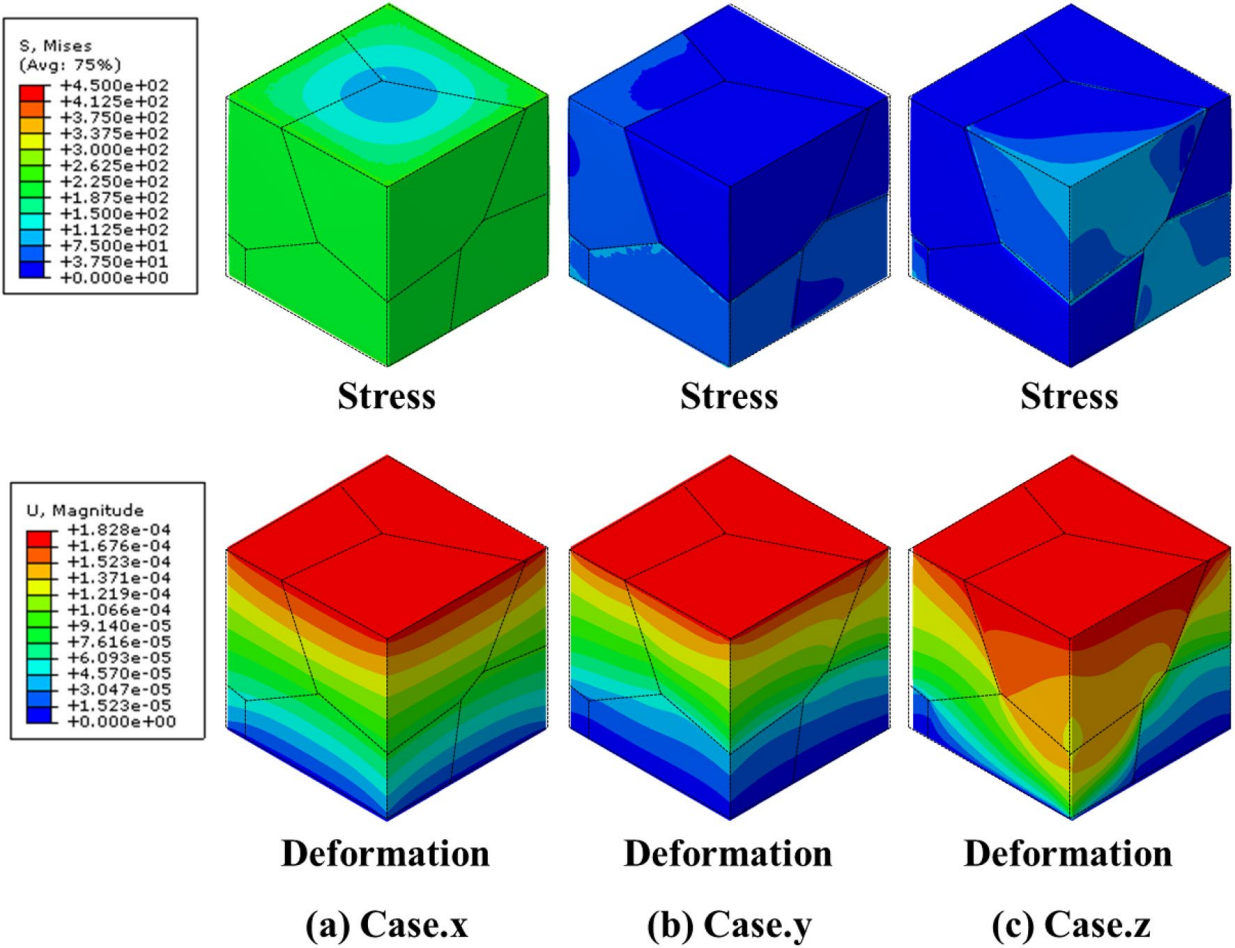
Stress and deformation FEM results of group 2.

Group 2 comprised cases “x”, “y”, and “z”, which had similar relative densities ranging from 27.41 to 30% but different Young’s modulus values ranging from 1.01 to 7.26 GPa. For case “x”, all the grain structures exhibited a relative density of 30%. This implied that the MGS exhibited the same mechanical behavior as the sheet-based gyroid with 30% relative density, as shown in Fig. 8a. For case “y”, the upper and lower grains had slight variations. The lower grains exhibited better mechanical properties. Hence, the lower grains primarily supported the entire MGS, whereas the upper grains demonstrated a more significant deformation under compressive loading, as shown in Fig. 8b. For case “z”, ${G}_{2}$, ${G}_{4}$, and ${G}_{6}$ had a relative density of 60%, whereas other grains had a relative density of 10%. Therefore, ${G}_{2}$ and ${G}_{4}$ primarily supported the entire MGS because the stress was transferred through those grains under compressive loading, as shown in Fig. 8c.

### Independent control of yield strength of MGS with respect to density

**Figure 9:**
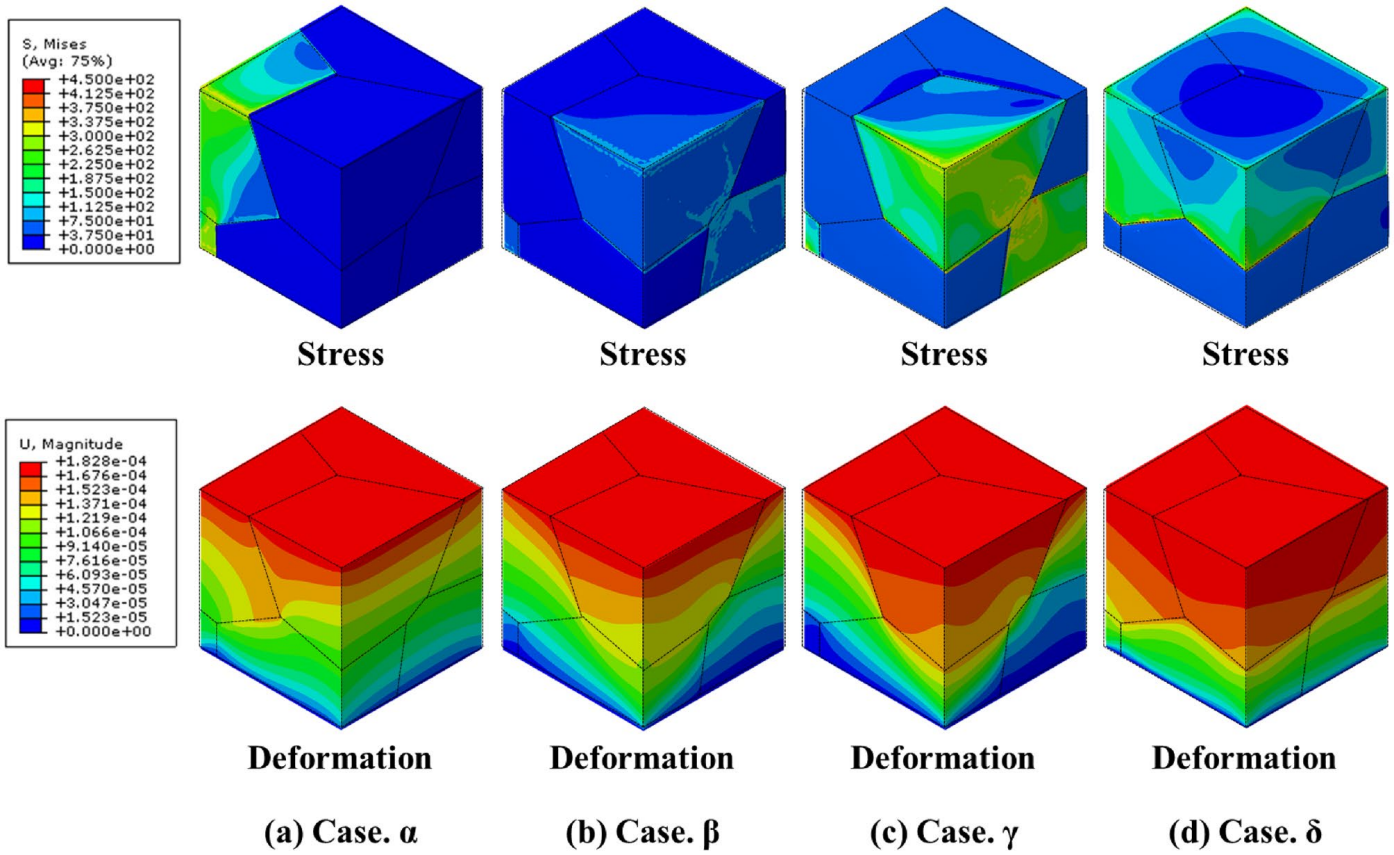
Stress and deformation FEM results of group 3.

The two groups of MGSs are marked as shown in Fig. 6b to explain the independent control of the yield strength of the MGS. Group 3 comprised points “$\alpha$”, “$\beta$”, “$\gamma$”, and “$\delta$”, whereas Group 4 comprised points “u”, “v”, and “w”, as shown in Fig. 6b. The relative density and yield strength of each MGS are listed in Table 4, and the specific relative densities of all the grains are listed in Table 5. In Group 3, the points had similar yield strengths ranging from 61.07 to 68.48 MPa, whereas the relative density ranged broadly from 27.96 to 57.86%. This implied that the relative density of an MGS could be controlled independently without changing the yield strength. For case “$\alpha$”, only ${G}_{3}$ and ${G}_{6}$ exhibited a relative density of 80%. Thus, they exhibited better mechanical properties than the other grains. Consequently, the two grains supported the total loading, as shown in Fig. 9a. For case “$\beta$”, ${G}_{2}$, ${G}_{4}$, and ${G}_{6}$ had a relative density of 50%; therefore, they were stronger than the other grains. Furthermore, because ${G}_{2}$ and ${G}_{4}$ were connected to each other, the total MGS was mainly supported by ${G}_{2}$ and ${G}_{4}$, as shown in Fig. 9b. For case “$\gamma$”, the arrangement of the relative densities of the grain structures was the same as that for case “$\beta$”; however, the former case exhibited better mechanical properties because its highest relative density was 80%, whereas that of the latter case was 50%. However, the yield strength values were similar in these cases. This was because both cases had a weak relative density of 20%; therefore, the overall mechanical property of the MGS was governed by these grains that possessed the weakest mechanical properties, as shown in Fig. 9c. For case “$\delta$”, the upper grain structure exhibited a relative density of 80%, whereas the lower grain structure had a value of 20%. The total relative density was the highest in Group 3, but the yield strength was similar to those of the other cases. This was because the overall mechanical properties were governed by the mechanical properties of the weakest grain, as shown in Fig. 9d.

**Table 4:** Relative densities and the corresponding yield strength of MGSs in Group 3 and Group 4.

Properties	$\alpha$	$\beta$	$\gamma$	$\delta$	u	v	w
Relative density (%)	27.96	31.66	43.33	57.86	57.86	56.67	57.52
Yield strength (MPa)	65.62	65.08	67.48	68.48	68.48	125.40	193.37

**Table 5:** Relative densities of MGS in Group 3 and Group 4.

Case	${G}_{1}$ (%)	${G}_{2}$ (%)	${G}_{3}$ (%)	${G}_{4}$ (%)	${G}_{5}$ (%)	${G}_{6}$ (%)	${G}_{Total}$ (%)
α	10	10	80	10	10	80	27.96
β	20	50	20	50	20	50	31.66
γ	20	80	20	80	20	80	43.33
δ	80	80	80	20	20	20	57.86
u	80	10	80	80	10	80	57.52
v	80	20	80	20	80	20	56.67
w	80	80	80	20	20	20	57.86

**Figure 10:**
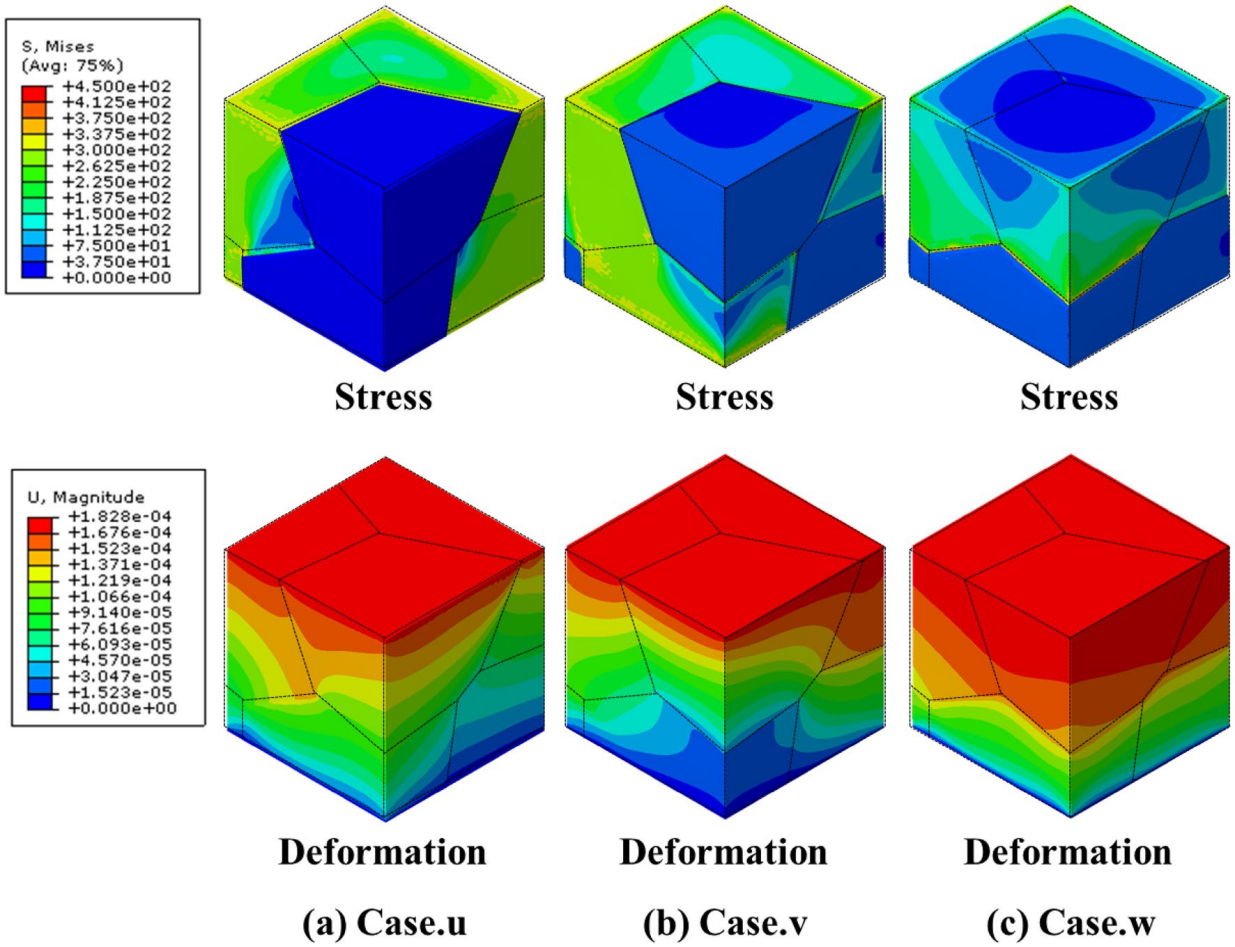
Stress and deformation FEM results of group 4.

In Group 4, the yield strength of each point ranged broadly from 68.48 to 193.37 MPa, but the relative density ranged from only 56.67% to 57.86%. This implied that the yield strength of the MGS could be customized while maintaining the relative density. For case “u”, ${G}_{1}$, ${G}_{3}$, ${G}_{4}$, and ${G}_{6}$ had a relative density of 80%, whereas ${G}_{2}$ and ${G}_{5}$ had a value of 10%. In particular, ${G}_{1}-{G}_{4}$ and ${G}_{3}-{G}_{6}$ were set as contact pairs and formed the upper and lower grain structures, respectively. Therefore, these two pairs exhibited the highest yield strength in the MGS, as shown in Fig. 10a. For case “v”, ${G}_{1}$, ${G}_{3}$, and ${G}_{5}$ had a relative density of 80%, which resulted in favorable mechanical properties. Among them, ${G}_{3}$ in the upper grain structure and ${G}_{5}$ in the lower grain structure formed the backbone of the structure. The yield strength of the MGS was determined by this contact, as shown in Fig. 10b. For case “w”, the upper grain structure had a relative density of 80%, whereas the lower grain structure had a value of 20%. Therefore, the total yield strength of the MGS was the lowest in Group 4 because of the worse mechanical properties of the lower grain structures, as shown in Fig. 10c. In conclusion, we could expand the designable area of the geometry in the same design space using the MGS. The mechanical properties of the MGS could be controlled through the interaction between the “grain structure” and “sheet-based gyroid lattice” in the MGS.

### ML-based regression for estimating mechanical properties of MGS

**Figure 11:**
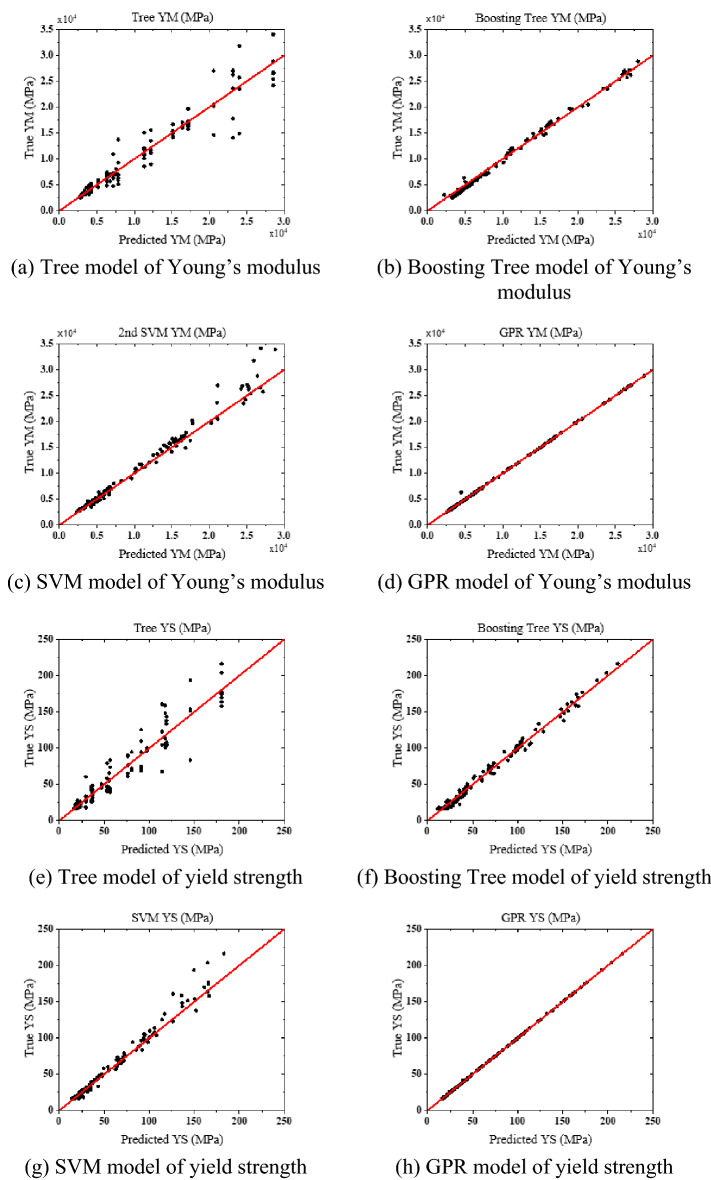
Predicted vs. true Young’s modulus and yield strength of MGS.

Multiple machine learning algorithms were tested in the MATLAB Regression Learner module, including the decision tree, boosting tree ensemble, SVM, and GPR, using the constructed database. The inputs for the regression models were the relative densities of all the grains in the MGS, and the outputs were the Young’s modulus and yield strength of the corresponding MGS. Figure 11 shows the predicted vs. actual values for the Young’s modulus and yield strength of the MGS. It can be noticed that the predictions by the machine learning models match the actual values quite well when the dots in the plot are located close to the line. This could be quantified by the R^2^ value, where the highest R^2^ value of 0.995 was obtained by GPR. The results showed that GPR was the most reliable regressor for estimating the Young’s modulus and yield strength of the MGS.

### Inverse design method for MGS with target mechanical property

Numerous applications of cellular materials have been suggested. In particular, the field of medical implants has an increasing demand for cellular materials. The key characteristics when designing the cellular structure for a medical implant include matching the stiffness of the cellular structure to that of the surrounding bones, ensuring the transferability of a load through the bone and implant, and alleviating the stress shielding effect^35^. In this study, we tested the possibility of using an MGS as a component of a medical implant. The target stiffness of the MGS was set at 30 GPa, which is the representative stiffness value for compact bone^36^. As mentioned in the previous section, the optimization technique based on PSO was used for the inverse design of the MGS.

**Figure 12:**
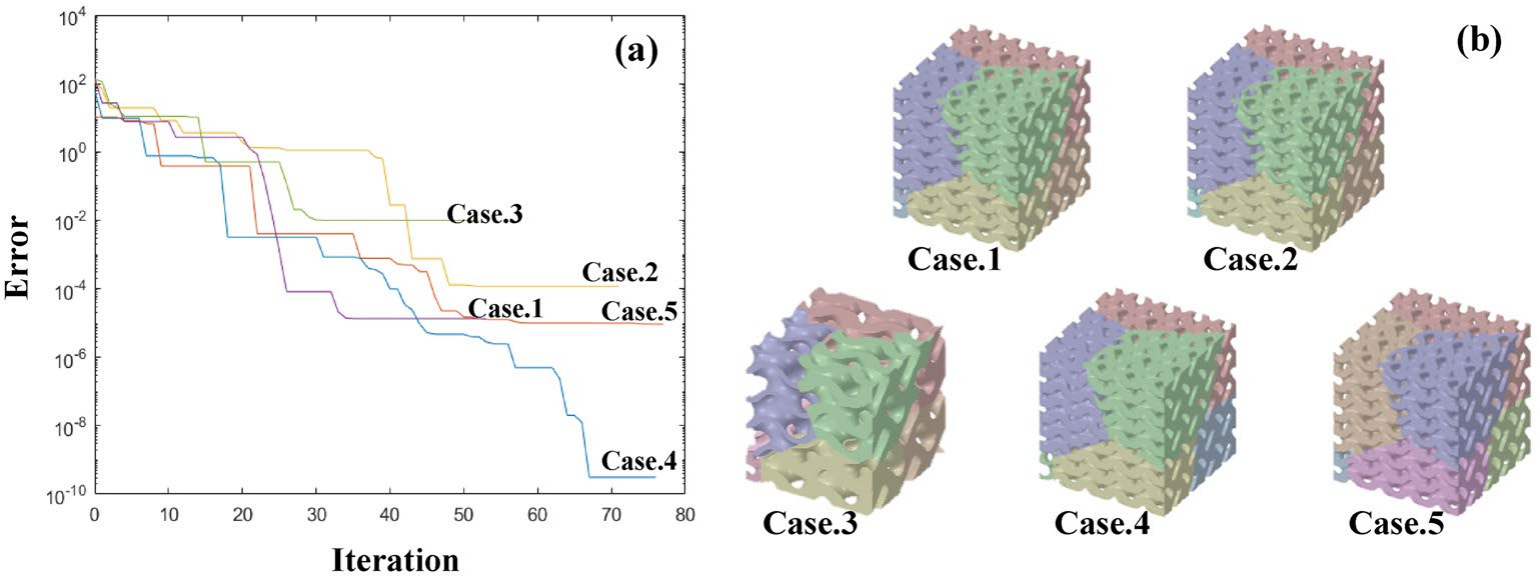
(**a**) Convergence of particle swarm algorithm in 5 individual initial conditions. (**b**) Optimized designs of MGS that satisfy the target young’s modulus.

The graph depicting the relative error vs. iteration number in Fig. 12a confirms the convergence of the PSO algorithm. The results are representative of 10 independent trials for the same objective function value, where outliers from the initial conditions of the particles were removed. The convergence speed changed based on the initial conditions, but all the cases converged within 70 iterations, indicating that the algorithm had reached the local suboptimal value. To confirm that the local suboptimal results satisfied our target performance (i.e., 30 GPa for the Young’s modulus of the MGS), we performed an FE analysis using the design parameters based on the PSO results. Table 6 shows a comparison between the PSO and FE results. As shown, the PSO yielded reliable results compared with the FE models, even though the suboptimal results were extracted from the PSO. The possibility of the PSO obtaining a suboptimal value could be an expansion of the design options for the MGS. Figure 12b shows the five MGSs corresponding to the convergence plot with the target performance. The results confirmed the design flexibility of the proposed design methodology, verifying the wide feasible range of mechanical properties offered by MGSs.

**Table 6:** Comparison between the PSO and FE result of Young’s modulus and yield strength.

Case	PSO result		FE result	
	Target Young’s modulus (GPa)	Yield strength (MPa)	Young’s modulus (GPa)	Yield strength (MPa)
1	30	187.47	29.44	180.53
2	30	191.73	29.96	191.01
3	30	192.94	29.34	182.20
4	30	190.22	29.63	176.45
5	30	190.15	30.09	189.76

## Conclusions

Inspired by polycrystalline structures, we proposed a novel structure (i.e., an MGS) composed of Voronoi cells and sheet-based gyroids, which enhanced the design flexibility and mechanical properties. Voronoi cells were generated through Voronoi tessellation using seeding points from the PPD and the SSI restriction algorithm. To increase the efficiency of the FE analysis, numerical homogenization was performed, wherein the mechanical properties of the sheet-based gyroid were defined as a function of the relative density. The deformation behaviors of 138 FE models with different relative densities were simulated to create a database for the MGS. This database was used to train an ML-based regression model that correlated the mechanical properties of the MGS with its relative density. The design parameters of the MGS that satisfied the target Young’s modulus of 30 GPa were obtained using PSO and the trained ML-based regression model. We believe that MGSs could be utilized in various applications such as medical implants, aircraft, and drones, in which high stiffness–weight ratios are desired.

The major conclusions of this study are as follows.

1. Grain structures inspired by polycrystalline structures were generated via Voronoi tessellation. This method made it possible to control the regularity of the grain structure by adjusting the seeding points using the PPD and SSI algorithm.
2. A systematic design methodology was developed using PSO to obtain the optimum MGS design that satisfied the target mechanical performance. Five design options with different relative densities were obtained using the proposed methodology.
3. The optimized MGS design was validated using the FE method. The perfect match between the estimated and simulated stiffness values of the MGSs confirmed the validity of the proposed design methodology and the potential of MGSs for use as components in medical implants.

## Supplementary Information

Supplementary Information.

## Data Availability

The datasets generated during the current study are available from the corresponding author on reasonable request.

## List of symbols

$E_{L}$: Young’s modulus of sheet-based gyroid lattice
$E_{B}$: Young’s modulus of base material
$\sigma_{L}$: Yield strength of sheet-based gyroid lattice
$\sigma_{B}$: Yield strength of base material
$C_{1}$: Proportionality constant for Young’s modulus
$C_{2}$: Proportionality constant for yield strength
$n$: Exponent for Young’s modulus
${\text{m}}$: Exponent for yield strength
$\rho^{*}$: Relative density of the sheet-based gyroids
$\upsilon_{L}$: Poisson’s ratio of sheet-based gyroid lattice
$p_{\text{n}}$: Proportionality constant for Poisson’s ratio
$G_{i}$: Mechanical properties of each grain
$x_{i}$: Vector of the design parameters
$d_{{\text{n}}}$: Density of each grain of the MGS
n: Number of grains
$$f$$: Objective function
$\underline {v}_{i}$: ith agent’s navigation vector
${\text{k}}$: Weight coefficient that models the inertia of the agent
t: Iteration step
$C_{3}$: Coefficient corresponding to the effects of the personal best experience and neighbor’s best experience pertaining to the velocity
$C_{4}$: Coefficient corresponding to the effects of the personal best experience and neighbor’s best experience pertaining to the velocity
$r_{1}$: Random value sampled from a uniform distribution
$r_{2}$: Random value sampled from a uniform distribution
$\underline {x}_{pbest,i}$: ith agent’s best cost function value
$\underline {x}_{leader}$: ith leader’s best cost function value
$G_{Total}$: Relative density of the MGS
